# Reduced-Dose or Discontinuation of Bevacizumab Might Be Considered after Variceal Bleeding in Patients with Hepatocellular Carcinoma Receiving Atezolizumab/Bevacizumab: Case Reports

**DOI:** 10.3390/medicina60010157

**Published:** 2024-01-15

**Authors:** Kyeong-Min Yeom, Young-Gi Song, Jeong-Ju Yoo, Sang Gyune Kim, Young Seok Kim

**Affiliations:** Department of Gastroenterology and Hepatology, Soonchunhyang University School of Medicine, Bucheon 14584, Republic of Korea; 121931@schmc.ac.kr (K.-M.Y.); syk5137@naver.com (Y.-G.S.); mcnulty@schmc.ac.kr (S.G.K.); liverkys@schmc.ac.kr (Y.S.K.)

**Keywords:** hepatocellular carcinoma, upper gastrointestinal bleeding, variceal bleeding, adverse event, bevacizumab

## Abstract

*Background and Objectives*: Variceal bleeding (VB) is the most concerning condition that is difficult to treat after atezolizumab/bevacizumab in patients with advanced hepatocellular carcinoma (HCC). *Materials and Methods*: We would like to introduce the cases of two patients who underwent bevacizumab reduction or discontinuation when VB occurred after atezolizumab/bevacizumab. *Results*: VB occurred in two patients who showed good tumor response after atezolizumab/bevacizumab treatment, and all VBs were successfully treated with endoscopic variceal ligations. In the first patient, VB did not occur as the tumor response decreased after a 50% reduction in bevacizumab. In the second patient, VB occurred again after a 50% bevacizumab reduction, so bevacizumab was discontinued and treatment with atezolizumab alone has been successfully maintained. *Conclusions*: Accordingly, we would like to suggest that considering bevacizumab dose reduction instead of changing to tyrosine kinase inhibitor may be a good clinical choice in atezolizumab/bevacizumab patients who develop VB.

## 1. Introduction

Hepatocellular carcinoma is often surgically inoperable at the time of diagnosis. Local treatment can be considered as a treatment option, but if the stage is high, systemic chemotherapy should be considered. A recent paradigm shift in chemotherapy resulted in the increased use of drugs for targeted therapy and immunotherapy in hepatocellular carcinoma (HCC) [1,2,3]. In 2020, a clinical study compared the effectiveness of treatment with sorafenib to treatment with atezolizumab and bevacizumab and showed the superiority of the latter option in terms of overall survival and progression-free survival [4]. Since then, the combination of atezolizumab and bevacizumab has been recommended as the first-line treatment in patients with advanced HCC [5].

Prior to broad clinical application, potential side effects of a treatment approach should be carefully examined in addition to its effectiveness. Bevacizumab was reported to be associated with the risk of increased blood pressure and bleeding, which may exacerbate portal hypertension and variceal bleeding in cirrhotic patients [6]. In a phase 2 trial of bevacizumab monotherapy for advanced HCC, the rate of variceal bleeding was reported to be up to 10% [7]. Based on this, patients with bleeding associated with portal hypertension or without effective prevention were excluded from the IMbrave 150 study. As a result, the rate of varices bleeding was 3.65% in the group treated with the combination of atezolizumab and bevacizumab, which was not higher than the reported variceal bleeding rate in previous studies, while it was 1.28% in the sorafenib group [4].

However, in actual clinical trials, somewhat higher levels of variceal bleeding are experienced than those reported in clinical trials. This may be because, unlike strict clinical trials, many patients with high bleeding risk are included in actual clinical practices.

If variceal bleeding occurs after using atezolizumab/bevacizumab, the treatment strategy is changed to a tyrosine kinase inhibitor such as sorafenib. However, in cases where tumor response is favorable with atezolizumab/bevacizumab, clinicians may be hesitant to switch to sorafenib, which is less effective than atezolizumab/bevacizumab. In this case report, we would like to introduce two cases in which variceal bleeding occurred in patients with an effective tumor response after using atezolizumab/bevacizumab and a good treatment effect was maintained by only reducing or discontinuing bevacizumab.

## 2. Materials and Methods

In South Korea, medical expenses for the combination of atezolizumab and bevacizumab to treat HCC have become eligible for reimbursement by the national health insurance system since May 2022 and this treatment has been increasingly used. During this period, atezolizumab/bevacizumab treatment was performed on a total of 34 advanced HCC patients at our hospital. Patients received 1200 mg of atezolizumab plus 15 mg per kilogram of body weight of bevacizumab intravenously every 3 weeks [4]. Among the patients, major gastrointestinal bleeding occurred in 5 patients (4 variceal bleeding, 1 peptic ulcer bleeding). Three out of five patients discontinued atezolizumab/bevacizumab treatment, and two patients reduced or discontinued bevacizumab. The cases introduced below are about two patients who reduced or discontinued the dose of bevacizumab and continued treatment with atezolizumab. The study protocol was approved by the Institutional Review Board of Soonchunhyang Bucheon Hospital (IRB No, 2022-11-011) and conformed to the ethical guidelines of the World Medical Association’s Declaration of Helsinki. Informed consent was waived from the IRB due to the retrospective design.

## 3. Results

**Case 1**

**Figure 1:**
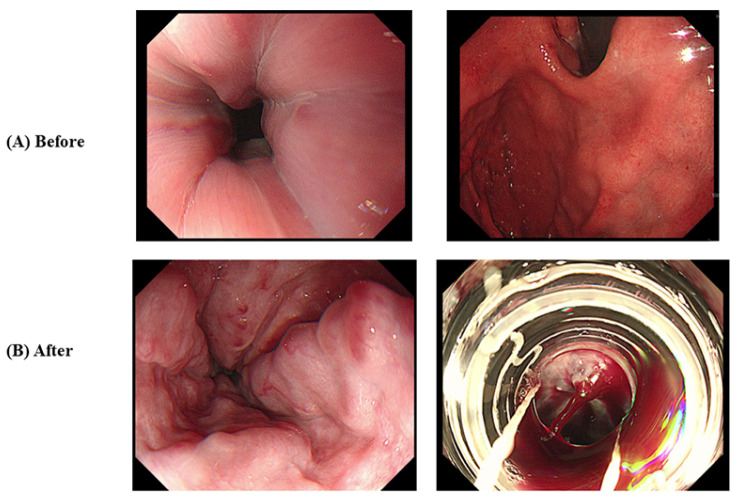
Endoscopic findings of Case 1. (**A**) Before atezolizumab/bevacizumab and (**B**) after atezolizumab/bevacizumab.

**Figure 2:**
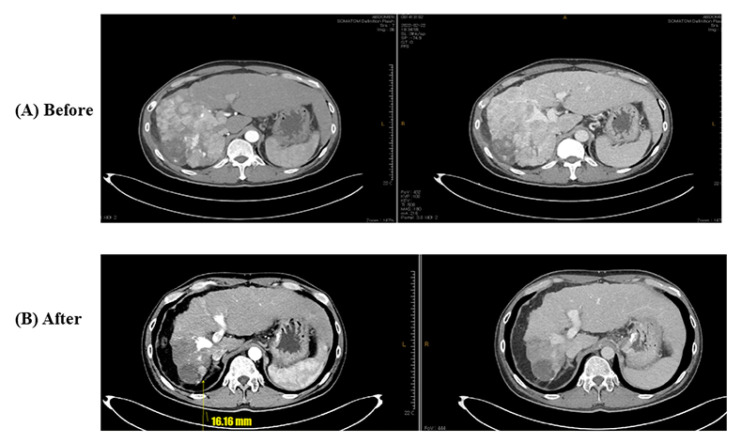
Radiologic findings of Case 1. (**A**) Before atezolizumab/bevacizumab and (**B**) after atezolizumab/bevacizumab.

**Figure 3:**
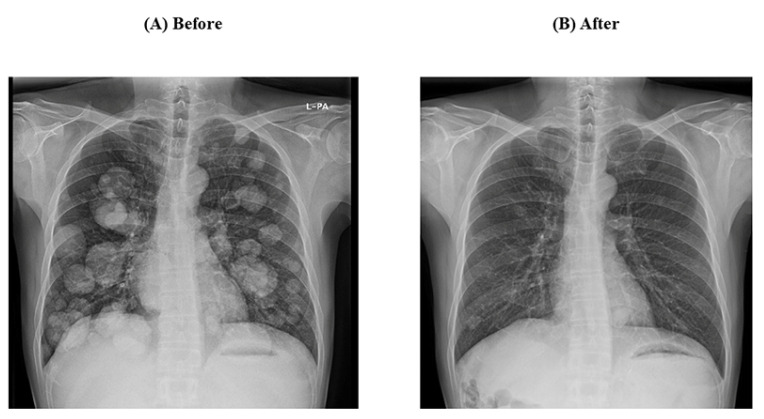
Improvement of lung metastasis of Case 1. (**A**) Before atezolizumab/bevacizumab and (**B**) after atezolizumab/bevacizumab.

A 50-year-old South Korean man was diagnosed with advanced HCC, Barcelona Clinic Liver Cancer (BCLC) stage C, and modified Union for International Cancer Control (mUICC) stage IVB, that had entirely invaded the right lobe and metastasized to both lungs and multiple lymph nodes. The patient was diagnosed with chronic hepatitis B 5 years ago, but was not treated, and tenofovir disoproxil fumarate was administered after the diagnosis of HCC and hepatitis B-related liver cirrhosis. Esophagogastroduodenoscopy (EGD) revealed grade I esophageal varices without red color sign (Li F1 Cw RC-) (Figure 1A). Abdominal computerized tomography (CT) detected hypervascular advanced HCC with a scant amount of ascites. His liver function was well preserved with Child–Pugh score of 6 with class A and Model for End-Stage Liver Disease (MELD) score of 7 and there was no history of other comorbidity or medication. The patient started treatment with atezolizumab and bevacizumab. After 4 cycles of atezolizumab and bevacizumab treatment, the patient visited the emergency room (ER) for melena and esophageal variceal bleeding was found on EGD. The size of the varix increased compared to the first examination and hemostasis was performed with endoscopic variceal ligation (EVL) (Figure 1B). Until this point, there was partial regression of HCC in response to chemotherapy. After that, an additional 2 cycles of atezolizumab and with bevacizumab reduction of 50% were performed. After 2 weeks from the 6th treatment with atezolizumab and bevacizumab, the patient again developed hematemesis due to variceal bleeding and it was treated with EVL. From then on, bevacizumab was discontinued and atezolizumab monotherapy was maintained. For preventing variceal bleeding, propranolol 40 mg bid was added. The size of HCC decreased over the course of chemotherapy. Currently, the patient is on the 23rd cycle with atezolizumab monotherapy, and intrahepatic lesions and lung metastasis have significantly improved compared to before treatment (Figure 2 and Figure 3). Variceal bleeding did not occur thereafter.

**Case 2**

**Figure 4:**
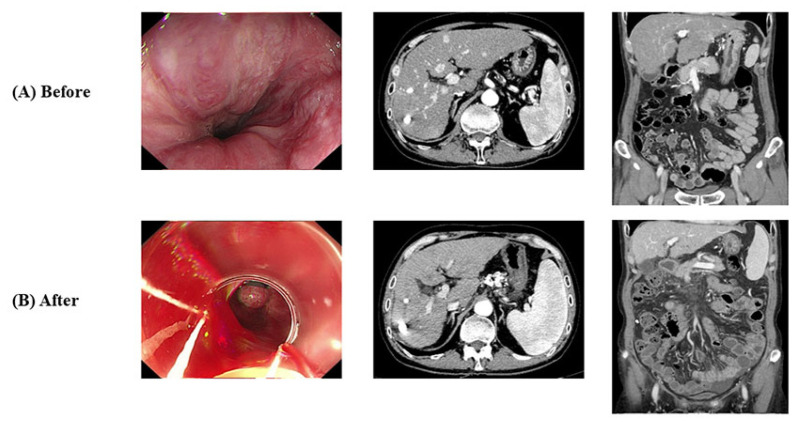
Endoscopic and radiologic findings of Case 2. (**A**) Before atezolizumab/bevacizumab and (**B**) after atezolizumab/bevacizumab.

A 54-year-old South Korean man was diagnosed with multinodular HCC. At the time of the HCC diagnosis, the patient was also diagnosed with cirrhosis due to chronic hepatitis B and antiviral drugs were administered. After 15 sessions of transcatheter arterial chemoembolization (TACE) over the 5-year period, the response of HCC was classified as progressive disease. Thus, he started treatment with atezolizumab and bevacizumab. HCC of the patient was classified as BCLC stage B and mUICC stage II. Before atezolizumab and bevacizumab treatment, grade I esophageal varices (Lm F1 Cw RC-) were revealed on EGD, Child–Pugh score was 6 with class A, and MELD score was 9. The patient had diabetes mellitus under insulin treatment but had no history of taking other medications causing bleeding tendency. Prior to atezolizumab and bevacizumab treatment, prophylactic EVL was performed twice. After prophylactic EVL, the patient started atezolizumab and bevacizumab treatment, and after the 3rd cycle of atezolizumab and bevacizumab treatment, he visited the ER for melena. EGD confirmed aggravation of esophageal varices (Lm F3 Cb RC+) with active bleeding, and therapeutic EVL was performed. For preventing variceal bleeding, carvedilol 6.25 mg bid was added. In abdominal CT, partial regression of HCC was noticed. After the 3rd cycle of atezolizumab and bevacizumab treatment, his liver function was well preserved with a Child–Pugh score of 6 with class A and MELD score of 9. Figure 4 presents the radiologic and endoscopic details. Three weeks after EVL, the patient received the 4th cycle of atezolizumab and bevacizumab treatment and bevacizumab was reduced to 50% due to bleeding risk. Currently, he is receiving the 19th atezolizumab/bevacizumab 50% reduction treatment, and the tumor response to date is partial response. Variceal bleeding did not occur thereafter.

## 4. Discussion

Through this study, we showed that when variceal bleeding occurs in patients showing good tumor response after using atezolizumab/bevacizumab, reducing or discontinuing bevacizumab may be a good option instead of changing to TKI. To date, case reports on bevacizumab dose reduction in hepatocellular carcinoma are very rare.

Bevacizumab is the first FDA-approved VEGF inhibitor that has been used in clinical practice for over 15 years. It is being used to treat various cancers such as colorectal cancer (CRC), non-small-cell lung cancer (NSCLC), RCC, and HCC. Meanwhile, hemorrhage, gastrointestinal (GI) perforation, and arterial thromboembolism are known as the most common and serious adverse effects of bevacizumab. We suppose that bevacizumab can increase the risk of bleeding, especially tumor-associated bleeding. Hemorrhage has been reported in 39.1% of patients with all cancers and in up to 44.2% of NSCLC patients [8], while GI bleeding has been reported in 24% of cancer patients [9]. The frequency of esophageal variceal bleeding in non-HCC cancer patients is not well known. In the IMbrave 150 study, the atezolizumab and bevacizumab combination therapy group reported higher GI bleeding than the sorafenib group (7% vs. 4.5%) and variceal bleeding (3.65% vs. 1.28%) [4]. However, the patients enrolled in this study were with well-controlled or well-treated varices, hence the risk of GI bleeding in this study sample was likely to be underestimated than that of the general patients with advanced HCC.

Jointly considered, we presume that the increased risk of bleeding associated with atezolizumab and bevacizumab treatment is likely due to bevacizumab. First, the inhibition of VEGF reduces the regenerative capacity of endothelial cells and decreases its function, increasing the risk of bleeding. Second, although the evidence is still insufficient, it is possible that inhibition of angiogenesis can reduce the proliferation of collateral vessels and increase portal hypertension, leading to esophageal varices bleeding [10,11]. Lastly, VEGF plays an important role in wound healing through angiogenesis and the use of bevacizumab may increase the possibility of bleeding while delaying the healing of EVL-induced ulceration in patients who have undergone EVL [12]. In actual clinical practice, several cases of varix aggravation or varix bleeding occurred after atezolizumab/bevacizumab treatment in patients with no varix or low-grade varix identified [13,14].

If variable bleeding occurs after using atezolizumab/bevacizumab, clinicians have to choose whether to maintain atezolizumab/bevacizumab, which is highly effective, or switch to a TKI with a lower risk of GI bleeding even if the effect is lower. When major GI bleeding occurs, switching to TKI is recommended, but related research or evidence is not high. A study related to bevacizumab dose reduction was conducted in Taiwan, and similar tumor responses were observed with standard dose bevacizumab (15 mg/kg) or low dose bevacizumab (7.5 mg/kg or fixed dose of 500 mg) [15]. In addition, there is a case series in which a 50% bevacizumab reduction was performed in vestibular schwannoma, and a favorable tumor response was observed with lower side effects [16]. However, considering atezolizumab monotherapy or bevacizumab reduction from the first cycle in patients with a high risk of variceal bleeding may result in poor tumor response. When comparing the atezolizumab/bevacizumab group and the atezolizumab-only group, overall survival was significantly shorter in the atezolizumab-only group at 5.6 months vs. 3.4 months. Some studies have shown that genomic expression can predict treatment response after atezolizumab/bevacizumab. High expression of VEGF receptor 2, Treg, myeloid inflammation, and TREM1/MDSC signatures was associated with longer progression-free survival in patients treated with atezolizumab/bevacizumab than in those treated with atezolizumab alone.

Thus, prevention of GI bleeding is necessary before atezolizumab and bevacizumab treatment. According to the Baveno VII consensus, a proactive primary prevention approach can be considered before atezolizumab and bevacizumab treatment such as the administration of non-selective beta blocker (NSBB) and prophylactic EVL [4]. However, in case 2 of the present study, variceal bleeding occurred even after prophylactic EVL and within 6 months of confirmation of grade I varix, warranting future research on more effective prevention methods. We propose that more frequent EGD screening would be useful. This may vary depending on the medical environment and facility, but considering the period during which the esophageal varices worsened in the patient in this case report, 6-month intervals might be necessary in high-risk patients [17]. In particular, it is important to identify the high-risk group for varix bleeding. A recent study identified age, platelet count, infiltrative tumor, and Child–Pugh score as high-risk predictors of varix bleeding for patients on atezolizumab/bevacizumab [18]. Based on this, it will also be necessary to strengthen surveillance or prophylactic procedures in high-risk groups.

Through this study, we found that varix bleeding is more commonly experienced in actual clinical practice than reported in clinical trials and we wanted to raise the topic of what treatment would be best in this situation, especially in patients showing good tumor response after atezolizumab/bevacizumab. There are still many unknown aspects regarding the prevention and treatment of variceal bleeding with atezolizumab/bevacizumab. Is prophylactic therapy helpful in the prevention of variceal bleeding after atezolizumab/bevacizumab? If EVL is performed, when will atezolizumab/bevacizumab treatment be possible? Is changing to TKI the only way for patients who experience varix bleeding after atezolizumab/bevacizumab? If the bevacizumab dose is to be reduced, what level of dose adjustment is appropriate? All of these questions are areas where future research should be conducted.

## 5. Conclusions

In conclusion, this study showed that, although it is a small number, in patients who showed good tumor response after atezolizumab/bevacizumab administration, atezolizumab monotherapy with discontinuation of bevacizumab can be a good option when variceal bleeding occurs.

## Data Availability

The datasets generated during and/or analyzed during the current study are available from the corresponding author upon reasonable request.
